# Antimicrobial activity of sulbactam-durlobactam against *Acinetobacter baumannii-calcoaceticus* complex strains causing non-respiratory and non-bloodstream infections from the United States (2023–2025)

**DOI:** 10.1128/spectrum.03289-25

**Published:** 2026-03-23

**Authors:** Elizabeth Cyr, Jill Argotsinger, Eric T. Beck, Robin R. Chamberland, Andrew E. Clark, Anne R. Daniels, Rachael Liesman, Mark Fisher, Philip Gialanella, Jonathan Hand, Amanda T. Harrington, Romney M. Humphries, Holly Huse, Robert Hamilton-Seth, Julia D. Hankins, Wesley D. Kufel, Scott W. Riddell, Meera Mehta, Ryan Demkowicz, A. Brian Mochon, Hossein Salimnia, Ayesha Khan, Virginia M. Pierce, Raghava Potula, Tsigereda Tekle, Patricia J. Simner, Robert J. Tibbetts, Christine Vu, Lilian M. Abbo, Octavio Martinez, Rebekah E. Dumm, Manohar B. Mutnal, Timothy C. Jenkins, Violeta Chávez, Paulette Pinargote, Jennifer Bowling, Erik Munson, Daniel V. Zurawski, David P. Nicolau, Tomefa E. Asempa, On behalf of the Acinetobacter baumannii Complex National Biorepository (ACNBio) Study Group

**Affiliations:** 1 Center for Anti-Infective Research and Development, Hartford Hospital 23893, Hartford, Connecticut, USA; 2 Advocate Lutheran General Hospital 21886, Chicago, Illinois, USA; 3 Department of Microbiology, ACL Laboratories 300396 , West Allis, Wisconsin, USA; 4 Department of Pathology, Saint Louis University School of Medicine 12274, , St. Louis, Missouri, USA; 5 Department of Pathology, University of Texas Southwestern Medical Center 12334, Dallas, Texas, USA; 6 Froedtert & Medical College of Wisconsin, Milwaukee, Wisconsin, USA; 7 Department of Pathology, Froedtert & Medical College of Wisconsin, Milwaukee, Wisconsin, USA; 8 Wisconsin Diagnostic Labs 655052 , Milwaukee, Wisconsin, USA; 9 Department of Pathology, University of Utah School of Medicine 12348 , , Salt Lake City, Utah, USA; 10 Associated Regional and University Pathologists Laboratories, Salt Lake City, Utah, USA; 11 Department of Pathology, Montefiore Medical Center, Albert Einstein College of Medicine, Bronx, New York, USA; 12 Department of Infectious Diseases, Ochsner Health, New Orleans, Louisiana, USA; 13 Loyola University Medical Center 25815, Maywood, Illinois, USA; 14 Department of Pathology, Microbiology and Immunology, Vanderbilt University Medical Center 12328, Nashville, Tennessee, USA; 15 Department of Pathology, Harbor-UCLA Medical Center 21640, Torrance, California, USA; 16 Department of Pathology and Laboratory Medicine, University of Kansas Medical Center, Kansas City, Kansas, USA; 17 State University of New York Upstate University Hospital, Syracuse, New York, USA; 18 Binghamton University School of Pharmacy and Pharmaceutical Sciences 465824, Binghamton, New York, USA; 19 State University of New York Upstate Medical University, Syracuse, New York, USA; 20 West Virginia University Hospitals 24037, Morgantown, West Virginia, USA; 21 Department of Pathology, West Virginia University School of Medicine 12355, Morgantown, West Virginia, USA; 22 Banner Health 3605, Phoenix, Arizona, USA; 23 Sonora Quest Laboratories 273991 , Phoenix, Arizona, USA; 24 Department of Pathology, University of Arizona College of Medicine, Phoenix, Arizona, USA; 25 Department of Pathology, Wayne State University School of Medicine 12267, Detroit, Michigan, USA; 26 Department of Pathology and Laboratory Medicine, UC Irvine School of Medicine, Irvine, California, USA; 27 Department of Pathology, University of Michigan Medical School 12266 , Ann Arbor, Michigan, USA; 28 Pathology and Laboratory Medicine, Temple University Health System, Philadelphia, Pennsylvania, USA; 29 Department of Pathology, Johns Hopkins University School of Medicine 1500 , Baltimore, Maryland, USA; 30 Pathology and Laboratory Medicine, Henry Ford Health, Detroit, Michigan, USA; 31 Department of Pharmacy, Jackson Health System 23214, Miami, Florida, USA; 32 Department of Medicine, University of Miami Miller School of Medicine 12235, Miami, Florida, USA; 33 Department of Pathology, University of Miami Miller School of Medicine 12235, Miami, Florida, USA; 34 Department of Pathology and Immunology, Washington University School of Medicine 12275 , St. Louis, Missouri, USA; 35 Department of Pathology and Laboratory Medicine, Baylor Scott & White Medical Center, Temple, Texas, USA; 36 Department of Medicine – Infectious Disease, Denver Health & Hospital Authority, Denver, Colorado, USA; 37 Department of Pathology and Laboratory Medicine, The University of Texas Health Science Center at Houston 12340, Houston, Texas, USA; 38 Division of Infectious Diseases, LSU-Ochsner Medical Center, Shreveport, Louisiana, USA; 39 Albany Medical Center 138334, Albany, New York, USA; 40 Department of Medical Laboratory Science, Marquette University 5505, Milwaukee, Wisconsin, USA; 41 Division of Infectious Diseases, Hartford Hospital, Hartford, Connecticut, USA; Houston Methodist Hospital, Houston, Texas, USA

**Keywords:** *Acinetobacter*, meropenem, CRAB, sulbactam-durlobactam

## Abstract

*Acinetobacter baumannii* most often causes pneumonia in critically ill patients. However, *A. baumannii* is also an important cause of a broader range of infections, including skin/wound and urinary tract infections. This study aims to evaluate the *in vitro* activity of sulbactam-durlobactam and comparator antibiotics, including meropenem and cefiderocol against *A. baumannii-calcoaceticus* complex isolates from non-respiratory and non-bloodstream sources. Samples included 285 *A. baumannii-calcoaceticus* complex isolates enriched for carbapenem resistance collected across 17 states in the United States (January 2023–May 2025). Antimicrobial susceptibility tests were conducted by manual broth microdilution and interpreted according to Clinical and Laboratory Standards Institute (CLSI) and Food and Drug Administration (FDA) (cefiderocol) standards. *A. baumannii* complex isolates were primarily cultured from skin/wound (58.6%), urinary tract (31.6%), and other sources (9.8%), including cerebrospinal and peritoneal fluid. Carbapenem resistance was observed in approximately 70% of isolates and more common among skin/wound cultures. Sulbactam-durlobactam was observed to be highly active (96.9% susceptible [S]; MIC_90_ 4 mg/L) and demonstrated greater activity than sulbactam (37.9% S; MIC_90_ 32 mg/L). Sulbactam-durlobactam also displayed high susceptibility rates across isolate sources, ranging from 96.4 (skin/wound) to 97.8% (urine). Cefiderocol demonstrated similar *in vitro* activity across culture sources and patient location, inhibiting >90 and >80% of isolates at CLSI and FDA susceptible breakpoints, respectively. Minocycline susceptibility was 69.1%, while tigecycline and eravacycline MICs_50/90_ were 1/4 and 0.5/1 mg/L, respectively. The observed data are consistent with results from surveillance studies among respiratory and bloodstream isolates and show that sulbactam-durlobactam demonstrates potent *in vitro* activity against clinical *A. baumannii* complex isolates from a variety of culture sources.

**IMPORTANCE:**

*Acinetobacter baumannii* is a difficult-to-treat pathogen that often affects hospitalized patients and is known for a high level of multi-drug resistance. While commonly associated with pneumonia, it also causes infections in wounds, the urinary tract, and other parts of the body. This study shows that sulbactam-durlobactam is highly effective against *A. baumannii* from various infection sites. The results are important because they can inform clinicians on the susceptibility profiles of *A. baumannii* from a variety of infection sources, not just lung and bloodstream. As new antibiotics come onto the market, it is important to continuously assess resistance patterns to inform patient and system-wide health decisions.

## INTRODUCTION

*Acinetobacter baumannii* is recognized by several global and national medical organizations, including the Infectious Diseases Society of America (IDSA), as a significant multidrug-resistant organism and a major public health threat due to its high mortality rates and potential to cause healthcare-associated outbreaks (1–4). While *A. baumannii* is most commonly associated with pneumonia and bloodstream infections, it is also capable of causing a broad spectrum of infections, including skin and soft tissue infections (SSTIs), urinary tract infections (UTIs), osteomyelitis, and meningitis (5–8). Notably, the pathogen has gained prominence as a key SSTI-associated organism among burn patients and military personnel injured in combat zones, particularly in Iraq and Afghanistan (8, 9). A recent 12-year retrospective analysis (2010–2021) of over 17,000 non-duplicate *Acinetobacter* spp. isolates from the United Arab Emirates reported urine (32.9%), sputum (29.0%), and soft tissue (25.1%) as the most common sources of isolation (10). These findings underscore the fact that the prevalence and distribution of infections caused by *Acinetobacter* spp. are diverse and influenced by geographic region, patient population, and clinical setting.

Antibiotic resistance in *Acinetobacter* spp. is mediated by the interplay of β-lactamase enzymes, aminoglycoside modifying enzymes, efflux pumps, and porins (11). Of these, the major resistance determinant is the expression of β-lactamases, including OXA-23 and OXA-24/40 that result in a carbapenem-resistant *A. baumannii* (CRAB) phenotype (11). (12), These mechanisms have eroded therapeutic options and led to the development of a novel β-lactam-β-lactamase inhibitor combination agent (sulbactam-durlobactam) to treat hospital-acquired bacterial pneumonia and ventilator-associated bacterial pneumonia caused by susceptible *A. baumannii-calcoaceticus* complex isolates (12, 13).

The *in vitro* activity of antimicrobial agents used to treat infections caused by *A. baumannii-calcoaceticus* complex isolated from non-respiratory and non-bloodstream infections has not been fully explored and is needed to elucidate antimicrobial resistance patterns within the United States. In this study, we evaluated the activity of sulbactam-durlobactam, meropenem, cefiderocol, and other antimicrobials against clinical strains from the ongoing *A. baumannii-calcoaceticus* Complex National Biorepository (ACNBio) program that allows the phenotypic and genotypic characterization of a diverse collection of clinical strains collected across U.S.

## MATERIALS AND METHODS

### Participating medical centers

The ACNBio Program was established to collect and characterize *A. baumannii-calcoaceticus* complex pathogens causing nosocomial infections via a U.S. network of sentinel hospitals. All sites received local institutional review board waivers prior to participation. Participating sites had the option of non-consecutive isolate collection to ease staff burden and could enrich for CRAB phenotype per local site definition. Sites provided de-identified data: year of collection, patient sex, age, location at time of culture (intensive care unit [ICU] versus non-ICU), and specimen source.

### Bacterial isolates

Unique *A. baumannii-calcoaceticus* complex isolates were collected from non-respiratory and non-bloodstream sources between January 2023 and May 2025. Isolates were identified by the microbiology laboratories of each participating medical center via conventional identification methods. Thereafter, isolates were transferred to trypticase soy agar (TSA) slants (Becton Dickinson, Sparks, MD) and shipped to the Center for Anti-Infective Research and Development (CAIRD) in Hartford, CT for susceptibility testing. Upon receipt at CAIRD, isolates were sub-cultured onto TSA supplemented with 5% sheep blood (Becton Dickinson, Sparks, MD) and preserved in skim milk at –80°C until antimicrobial susceptibility testing was performed.

### Antimicrobial susceptibility

All the drugs tested in this study have been listed as potential therapeutic agents to treat CRAB infections in the IDSA antimicrobial resistance expert guidance and include agents from representative antibiotic classes, such as cephalosporins, carbapenems, polymyxins, and tetracyclines (1). Ampicillin (MedChemExpress #81549), sulbactam (USP #R03980), meropenem (Sigma Aldrich #LRAD1014), cefiderocol (Fetroja commercial vial #0021), colistin (MedChemExpress #62961), tigecycline (Sigma Aldrich #LRAC6890), and minocycline (Sigma Aldrich #191077) antibiotic powders were purchased. Durlobactam (Corden Pharma #138677) and eravacycline (Tetraphase #0026000135) powder were supplied by Innoviva Specialty Therapeutics. MIC broth trays were prepared with a Biomek 3000 (Beckman Instruments, Inc., Fullerton, California) across an MIC range specific to each antibiotic. Susceptibility tests were conducted by manual broth microdilution and interpreted according to CLSI standards (14, 15). Sulbactam MICs were interpreted using the sulbactam MIC component of ampicillin/sulbactam (CLSI breakpoint 8/4 mg/L) and thus assessed with a sulbactam susceptibility breakpoint of 4 mg/L per CLSI standards. Cefiderocol MICs were assessed with both CLSI (4 mg/L) and FDA (1 mg/L) susceptibility breakpoints. *A. baumannii* NCTC 13304, *Escherichia coli* ATCC 25922, and *Pseudomonas aeruginosa* ATCC 27853 were used as quality control strains for antimicrobial testing and run in parallel with test isolates on each day.

### Carbapenemase screening

Carbapenemase contribution to reduced susceptibility was investigated; isolates with sulbactam-durlobactam MICs ≥ 8 mg/L underwent genotypic carbapenemase testing on the Xpert Carba-R assay (GXCARBAR-10; Cepheid, Sunnyvale, CA).

### Analysis

Demographic data were assessed using descriptive statistics, including percentages for categorical data. Susceptibility rates (percentage of susceptible isolates within the total number of isolates), MIC_50_, and MIC_90_ were computed using Microsoft Excel and presented for each antibiotic, where applicable.

## RESULTS AND DISCUSSION

A total of 285 isolates were received from contributing sites and evaluated in this study. The demographic data of the isolates are presented in Table 1. The median patient age was 62 years, and the majority (64.6%) were male. Approximately 60% of isolates were obtained from skin/wound and 31.6% from the urinary tract. Other sources included body fluids (e.g., peritoneal fluid, cerebrospinal fluid, as well as bone culture).

**TABLE 1:** Demographics and characteristics of the study population

Description	Population (*N* = 285)
Age, median (IQR), years	62 (48–72)
Male sex, n (%)	184 (64.6%)
Site location^*a*^	
Northeast	28 (9.8%)
South	129 (45.3%)
Midwest	74 (25.9%)
West	54 (19%)
Patient location	
ICU	81 (28.2%)
Medical ICU	33
Surgical ICU	10
Other ICU^*b*^	38
Non-ICU	194 (67.6%)
NA	12 (4.2%)
Culture source	
Skin/wound	167 (58.6%)
Urine	90 (31.6%)
Other source^*c*^	28 (9.8%)

^*a*^ U.S. Census Bureau Regional Divisions, ICU: intensive care unit, and NA: not available.

^*b*^ Other ICU: burn, neurology, cardiac.

^*c*^ Other source: peritoneal fluid, cerebrospinal fluid, synovial fluid, bone culture.

The results of antimicrobial susceptibility testing and MIC_50/90_ values are shown in Table 2. Antimicrobial susceptibilities ranged from 30.2% with meropenem to 96.9% with sulbactam-durlobactam, the most active agent. ICU status and culture source had an impact on meropenem non-susceptibility (MEM-NS) rates. MEM-NS was numerically higher among isolates recovered from skin/wound (74.9%) compared to urinary tract isolates (60.0%). Similarly, a larger proportion of MEM-NS isolates were observed in ICU patients (85.2%) relative to their non-ICU counterparts (61.5%).

**TABLE 2:** Antimicrobial susceptibility rates and MIC_50/90_ values of *Acinetobacter baumannii-calcoaceticus* complex isolates

Category	Antimicrobial agent	%S	%I	%R	MIC_50_ (mg/L)	MIC_90_ (mg/L)
All isolates (*n* = 285)	Sulbactam^*a*^	37.9	12.6	49.5	8	32
	Sulbactam-durlobactam	96.9	1.7	1.4	1	4
	Meropenem	30.2	1.0	68.8	32	>64
	Cefiderocol_CLSI_	94.8	0.7	4.5	0.25	4
	Cefiderocol_FDA_	81.7	8.1	10.2	0.25	4
	Colistin	–^*d*^	94.4	5.6	0.5	2
	Tigecycline^*b*^	–	–	–	1	4
	Eravacycline^*b*^	–	–	–	0.5	1
	Minocycline	69.1	26.7	4.2	2	8
Skin/wound isolates (*n* = 167)	Sulbactam^*a*^	37.7	12.0	50.3	16	32
	Sulbactam-durlobactam	96.4	1.8	1.8	2	4
	Meropenem	25.1	0.6	74.3	64	>64
	Cefiderocol_CLSI_	95.8	1.2	3.0	0.25	4
	Cefiderocol_FDA_	78.4	10.8	10.8	0.25	4
	Colistin	–	94.6	5.4	0.5	2
	Tigecycline^*b*^	–	–	–	1	2
	Eravacycline^*b*^	–	–	–	0.5	1
	Minocycline	65.3	31.1	3.6	4	8
Urine isolates (*n* = 90)	Sulbactam^*a*^	42.2	10.0	47.8	8	32
	Sulbactam-durlobactam	97.8	1.1	1.1	1	4
	Meropenem	40.0	2.2	57.8	32	>64
	Cefiderocol_CLSI_	94.4	0.0	5.6	0.25	2
	Cefiderocol_FDA_	87.8	3.3	8.9	0.25	2
	Colistin	–	92.2	7.8	0.5	2
	Tigecycline^*b*^	–	–	–	1	2
	Eravacycline^*b*^	–	–	–	0.5	1
	Minocycline	72.2	22.2	5.6	2	8
Other sources (*n* = 28)^*c*^	Sulbactam^*a*^	25.0	25.0	50.0	8	32
	Sulbactam-durlobactam	96.4	3.6	0	1	4
	Meropenem	28.6	0	71.4	32	>64
	Cefiderocol_CLSI_	89.3	0	10.7	0.5	>35
	Cefiderocol_FDA_	82.2	7.1	10.7	0.5	>32
	Colistin	–	100	0	0.5	0.5
	Tigecycline^*b*^	–	–	–	2	4
	Eravacycline^*b*^	–	–	–	0.5	1
	Minocycline	82.1	14.3	3.6	2	8
ICU isolates (*n* = 81)	Sulbactam^*a*^	23.5	16.0	60.5	16	32
	Sulbactam-durlobactam	98.8	0	1.2	2	4
	Meropenem	14.8	0.0	85.2	64	128
	Cefiderocol_CLSI_	92.6	0.0	7.4	0.5	4
	Cefiderocol_FDA_	77.8	13.6	8.6	0.5	4
	Colistin	–	96.3	3.7	0.5	1
	Tigecycline^*b*^	–	–	–	2	2
	Eravacycline^*b*^	–	–	–	0.5	1
	Minocycline	60.5	34.6	4.9	4	8
Non-ICU isolates (*n* = 192)	Sulbactam^*a*^	45.3	12.0	42.7	8	32
	Sulbactam-durlobactam	95.8	2.6	1.6	1	4
	Meropenem	38.5	1.6	59.9	32	128
	Cefiderocol_CLSI_	95.3	1.0	3.6	0.25	4
	Cefiderocol_FDA_	83.9	5.2	10.9	0.25	4
	Colistin	–	93.2	6.8	0.5	2
	Tigecycline^*b*^	–	–	–	1	2
	Eravacycline^*b*^	–	–	–	0.5	1
	Minocycline	76.0	19.8	4.2	1	8
Sulbactam non-susceptible isolates (*n* = 346)	Sulbactam^*a*^	0	20.3	79.7	16	32
	Sulbactam-durlobactam	94.9	2.8	2.3	2	4
	Meropenem	4.5	1.1	94.4	64	128
	Cefiderocol_CLSI_	93.8	0.6	5.6	0.5	4
	Cefiderocol_FDA_	76.3	11.3	12.4	0.5	4
	Colistin	–	94.9	5.1	0.5	1
	Tigecycline^*b*^	–	–	–	2	4
	Eravacycline^*b*^	–	–	–	0.5	1
	Minocycline	57.6	35.6	6.8	4	8
Carbapenem non-susceptible isolates (*n* = 402)	Sulbactam^*a*^	15.1	16.1	68.8	16	32
	Sulbactam-durlobactam	95.5	2.5	2.0	2	4
	Meropenem	0	1.5	98.5	64	128
	Cefiderocol_CLSI_	93.5	1.0	5.5	0.5	4
	Cefiderocol_FDA_	75.3	11.1	13.6	0.5	4
	Colistin	–	94.0	6.0	0.5	2
	Tigecycline^*b*^	–	–	–	2	4
	Eravacycline^*b*^	–	–	–	0.5	1
	Minocycline	55.8	38.2	6.0	4	8
Cefiderocol_FDA_ non-susceptible isolates (*n* = 125)	Sulbactam^*a*^	19.2	21.2	59.6	16	64
	Sulbactam-durlobactam	94.3	3.8	1.9	2	4
	Meropenem	5.8	0	94.2	32	128
	Cefiderocol_FDA_	0	44.2	55.8	4	64
	Colistin	–	88.5	11.5	0.5	4
	Tigecycline^*b*^	–	–	–	2	4
	Eravacycline^*b*^	–	–	–	0.5	1
	Minocycline	78.8	21.2	0	2	8

^*a*^ Sulbactam MICs are interpreted using the sulbactam component of ampicillin-sulbactam MIC breakpoints (≤8/4 [susceptible], 16/8 [intermediate], ≥32/16 [resistant]) per CLSI M100 (Ed35; 2025).

^*b*^ MIC interpretative criteria are not published by CLSI M100 (Ed35; 2025) for tigecycline and eravacycline against *Acinetobacter* spp.

^*c*^ Other sources included body fluids (e.g., peritoneal fluid, cerebrospinal fluid as well as bone culture).

^*d*^ "–" means Not applicable.

Very few studies focused on *Acinetobacter* have evaluated antimicrobial *in vitro* activity according to culture source and/or ICU status. A retrospective *in vitro* study (January 2017 to December 2017) by McCann et al. utilizing a U.S. hospital database yielded several insights (16). A total of 312,075 isolates were evaluated, with *Enterobacterales* comprising a majority (85.2%; *n* = 265,781) and *Acinetobacter* spp. (1.1%; *n* = 3,414) as the smallest isolate group. Urine and skin/wound were the predominant sources at 59.4 and 15.6%, respectively, of the total data set. The overall carbapenem non-susceptible rate was 3.4%. However, among just the *Acinetobacter* spp., approximately one in three isolates (35.6%) were carbapenem non-susceptible (16). Notably, the contributions of different pathogens to carbapenem-NS rates varied depending on the isolate source. For example, the contribution of *Acinetobacter* spp. to carbapenem-NS isolates was ≤5% for urine and intra-abdominal sources but accounted for approximately 20 and 15% of carbapenem-NS isolates for skin/wound and blood, respectively (16). Similar to our findings, ICUs had significantly higher rates of carbapenem non-susceptibility than non-ICU settings in the McCann study (16, 17).

In the present study, the addition of durlobactam to sulbactam lowered the MIC_90_ 3-doubling dilutions from 32 to 4 mg/L. Sulbactam-durlobactam maintained high activity against sulbactam-NS (94.9%) and carbapenem-NS (95.5%) isolates (Table 2). The percent susceptible to sulbactam-durlobactam did not differ across isolate categories: ICU (98.8%), non-ICU (95.8%), skin/wound (96.4%), urine (97.8%), and other sources (96.4%). Previously published reports have also described the *in vitro* activity of sulbactam-durlobactam across various infection sources (18, 19). A global study (2016 to 2021) characterized 5,032 *A. baumannii-calcoaceticus* complex isolates from 33 countries. The infection source breakdown for that study was: lower respiratory (54.3%), bloodstream (20.2%), urinary tract (16.5%), skin/soft tissue (4.5%), and intra-abdominal (4.3%). Excluding respiratory and bloodstream sources, antimicrobial susceptibilities between urinary tract and skin/soft tissue isolates were similar; in particular, meropenem and sulbactam-durlobactam susceptibility rates were 61.2–61.7% and 98.9–99.1%, respectively (19). These data support the consistent pattern of potent (>90% susceptibility) sulbactam-durlobactam activity observed among contemporary U.S.-specific isolates in the current study. Given the option of non-consecutive isolate submission into our surveillance study, susceptibility rates should be interpreted in the context of the enriched CRAB phenotype that comprised this data set relative to prevalence rates in other studies.

Nine isolates (3.2%) demonstrated sulbactam-durlobactam MIC values ≥ 8 mg/L over the surveillance period. These isolates were primarily from non-ICU patients (eight out of nine) and skin/wound sources (six out of nine). Genotyping revealed two out of the nine isolates encoded genes for New Delhi metallo-β-lactamase (NDM). Similar studies have elucidated NDM and penicillin binding protein 3 (PBP3) alterations as the main resistance mechanisms contributing to reduced *A. baumannii* susceptibility to sulbactam-durlobactam. Notably, NDM results in hydrolysis of sulbactam, while PBP3 alterations result in reduced sulbactam affinity to the essential PBP through which it exerts its antimicrobial effect (17, 20).

Cefiderocol also retained consistent activity (MIC_50_: 0.25 mg/L; MIC_90_: 4 mg/L), regardless of ICU status and culture source, with susceptibilities ranging from 89.3 to 95.8% per CLSI breakpoints; however, the different susceptibility breakpoint set by the FDA results in substantially lower susceptibility rates (77.8–87.8%). Of the three tetracyclines evaluated in this study, only minocycline has evaluable CLSI breakpoints, with approximately 70% of all isolates categorized as susceptible. Although tigecycline and eravacycline lack established CLSI breakpoints, they demonstrated potent *in vitro* activity, inhibiting 90% (MIC_90_) of isolates at MICs of 4 and 1 mg/L, respectively. As a whole, resistance rates and MIC_90_ values trended higher for the tetracyclines against *A. baumannii* isolated from the ICU and skin/wound cultures relative to non-ICU and urine cultures. For colistin, in the absence of a CLSI susceptible breakpoint, 94.4% of isolates were categorized as intermediate, and this rate remained consistent across infection source and ICU status.

In conclusion, *A. baumannii* from non-respiratory and non-bloodstream infection sources was highly susceptible (>95%) to sulbactam-durlobactam. Despite elevated ampicillin-sulbactam and carbapenem resistance rates, sulbactam-durlobactam non-susceptibility and MBL-harboring *A. baumannii* were rare in this U.S. surveillance data set. These data underscore the importance of active surveillance regardless of infection source or critical care status.
